# Clinical remission and subsequent relapse in patients with juvenile idiopathic arthritis: predictive factors according to therapeutic approach

**DOI:** 10.1186/s12969-021-00607-0

**Published:** 2021-08-21

**Authors:** Mireia Castillo-Vilella, Nuria Giménez, Jose Luis Tandaipan, Salvador Quintana, Consuelo Modesto

**Affiliations:** 1 Department of Rheumatology, Hospital UniversitariSagrat Cor, C/ Londres, 28-38 3rd floor, 08029 Barcelona, Spain; 2 Department of Rheumatology, Hospital UniversitariMútua Terrassa, Terrassa, Spain; 3 Department of Rheumatology, Pediatric Rheumatology Unit, Hospital UniversitariValld’Hebrón, Barcelona, Spain; 4 Departament de Farmacologia, Terapèutica i Toxicologia, UniversitatAutònoma de Barcelona, Barcelona, Spain; 5 Research Unit, University Hospital of Mútua Terrassa, Research Foundation Mútua Terrassa, Universitat de Barcelona, Barcelona, Spain; 6 Hospital Sant Jaume de Calella, Laboratori de Referència de Catalunya i Corporació de Salut del Maresme i la Selva, Barcelona, Spain; 7 Departament of Rheumatology and Systemic Autoimmune Diseases, Hospital de la Santa Creu i Sant Pau, Universitat Autònoma de Barcelona, Barcelona, Spain; 8 Departament de Medicina, Universitat Autònoma de Barcelona, Barcelona, Spain; 9 Retired, Barcelona, Spain; 10 Department of Rheumatology, Hospital Universitario de Cruces, Barakaldo, Spain

**Keywords:** Juvenile arthritis, Remission, DMARD, Withdrawal, Relapse, Predictor

## Abstract

**Background:**

Juvenile idiopathic arthritis constitutes a significant cause of disability and quality of life impairment in pediatric and adult patients. The aim of this study was to ascertain clinical remission (CR) and subsequent relapse in juvenile idiopathic arthritis (JIA) patients, according to therapeutic approach and JIA subtype. Evidence in literature regarding its predictors is scarce.

**Methods:**

We conducted an observational, ambispective study. Patients diagnosed of JIA, treated with synthetic and/or biologic disease modifying antirheumatic drugs (DMARD) were included and followed-up to December 31st, 2015. Primary outcome was clinical remission defined by Wallace criteria, both on and off medication. In order to ascertain CR according to therapeutic approach, DMARD treatments were divided in four groups: 1) synthetic DMARD (sDMARD) alone, 2) sDMARD combined with another sDMARD, 3) sDMARD combined with biologic DMARD (bDMARD), and 4) bDMARD alone.

**Results:**

A total of 206 patients who received DMARD treatment were included. At the time the follow-up was completed, 70% of the patients in the cohort had attained CR at least once (144 out of 206), and 29% were in clinical remission off medication (59 out of 206).

According to treatment group, CR was more frequently observed in patients treated with synthetic DMARD alone (53%). Within this group, CR was associated with female sex, oligoarticular persistent subtypes, ANA positivity, Methotrexate treatment and absence of HLA B27, comorbidities and DMARD toxicity. 124 DMARD treatments (62%) were withdrawn, 64% of which relapsed. Lower relapse rates were observed in those patients with persistent oligoarticular JIA (93%) when DMARD dose was tapered before withdrawal (77%).

**Conclusions:**

More than two thirds of JIA patients attained CR along the 9 years of follow-up, and nearly one third achieved CR off medication. Females with early JIA onset, lower active joint count and ANA positivity were the ones achieving and sustaining remission more frequently, especially when receiving synthetic DMARD alone and in the absence of HLA B27, comorbidities or previous DMARD toxicity.

## Background

Juvenile idiopathic arthritis is the most common chronic rheumatic disease in childhood. This term comprises an heterogenous group of arthritis of unknown aetiology, each of which has differential genetics, etiopathogenesis, onset age and disease outcomes [1, 2]. It constitutes a significant cause of disability and quality of life impairment in JIA pediatric and adult patients [3–5].

Recent advances in JIA treatment addressing more specific targets have led to better short and long-term disease outcomes. Evidence throughout the last decade has shown that an early and tight treatment to target approach of the disease increases the likelihood of achieving and sustaining clinical remission over time [6–9]. Nevertheless, a notable proportion of patients relapse, either while still on medication or after its withdrawal [10–12].

On the other hand, it seems reasonable to withdraw all medications in those patients that already have achieved CR to avoid the costs and possible adverse events derived of maintaining it over time. In this scenario, the main concern to most physicians is the occurrence of a relapse, which has shown to be frequent in most studies [13–15]. In those patients who attain CR despite having a more aggressive course of the disease, maintaining tapered DMARD doses could increase the likelihood of sustaining a quiescent status of the disease over time, and avoid relapse after treatment withdrawal [9, 13, 15, 16].

No consistent evidence or guidelines are available regarding how or when to withdraw DMARD, or whether to do it abruptly or after the dose has been gradually tapered. Some studies have analysed the possible predictors of flare in patients in CR on and off medication, obtaining heterogeneous and conflicting results. Moreover, those predictors have not been validated to address therapeutic strategies in a real practice setting. This fact is partly explained by the heterogeneity within JIA categories, which consequently generates great variability in the definition and composition of study populations. In addition, evidence in literature has shown heterogeneity in clinical remission and inactive disease definitions, as well as significant variability in therapeutic strategies carried out by pediatric rheumatologists in JIA treatment [13, 15, 17, 18].

Few studies have analysed the prevalence and likelihood of CR regarding the therapeutic approach, which stands the aim of our study. Administering synthetic and biologic DMARD alone or combined is of significant relevance due to its influence on its tolerability, adherence, safety, efficacy and on subsequent disease outcomes [9, 19, 20].

## Methods

We conducted a single-center, observational, retrospective-prospective study of JIA patients who were attended at Vall d’Hebrón University Hospital’s Pediatric Rheumatology Unit. This tertiary care hospital is a reference centre for JIA patients in Catalonia (Spain), where the mean JIA incidence was estimated to be of 6.9 per 100,000 children by 2010 [21].

A retrospective cohort was created on January 1st, 2012, in which patients diagnosed of JIA, treated with DMARD, and followed-up to December 31st, 2013, were included. Thereafter, a two-year prospective follow-up was performed from January 1st, 2014 to December 31st, 2015.Patients were included if: (a) the symptoms started before age 16 and lasted for at least 6 weeks, (b) fulfilled the ILAR Edmonton 2001 criteria [22], and (c) were receiving synthetic and/or biologic DMARD.

Patients were evaluated every 3 months according to study protocol and local clinical practice. Demographic, clinical, immunologic and treatment data were collected from paper charts and electronic medical records. Uveitis and other non-uveitis comorbidities were considered as separate variables taking into account the clinical relevance of JIA-associated uveitis. Non-uveitis comorbidities were defined as coexisting disorders or diseases, other than uveitis, and not causally related to drug toxicity. DMARDs were initiated in therapeutic doses according to patient’s body surface when an optimal control of disease activity could not be attained or when corticosteroids could not be withdrawn along the first 3 months from disease or follow-up onset. Treatment adjustments were performed at scheduled appointments or earlier, if needed, in flare occurrence. DMARD tapering was defined as performing either a dose reduction (below therapeutic dose) or an increase in the interval between doses. When dose tapering was not performed, the DMARD was abruptly withdrawn.

This study was done in accordance with Ethics Research Committees of Vall d’Hebrón University Hospital and Mútua Terrassa University Hospital, in the corresponding phases of the study. Written consent was obtained from patients and/or parents.

### Clinical remission

Primary outcome was Clinical Remission (CR) of the disease, defined by Wallace criteria [23, 24] as: (a) no joints with active arthritis, (b) no fever, rash, serositis, splenomegaly or lymphadenopathy attributable to JIA, (c) no active uveitis, (d) Normal erythrocyte sedimentation rate or C-reactive protein level, (e) lowest possible physician’s global assessment of disease activity score, and (f) morning stiffness shorter than 15 min. CR on medication was defined as criteria were fulfilled for at least 6 months. CR off medication was defined as criteria were fulfilled for at least 12 months after all medication was withdrawn [23, 24]. Relapse was defined as no longer fulfilling criteria at one or more visits. To analyse CR according to the therapeutic approach, treatments with DMARD were divided in four groups: 1) synthetic DMARD (sDMARD) alone, 2) sDMARD combined with another sDMARD, 3) sDMARD combined with biologic DMARD (bDMARD), and 4) bDMARD alone. Secondary outcome measures were CR predictive factors, DMARD dose tapering, tapering method and duration, DMARD withdrawal due to CR, treatment duration up to withdrawal, relapse after withdrawal, time to relapse and relapse predictive factors. All patients withdrawing any DMARD treatment due to CR fulfilled at least Wallace’s criteria of CR on medication.

### Statistical analysis

Qualitative variables were expressed as absolute values and percentages. The normality was explored with the Kolmogorov-Smirnov test. Considering that all study variables presented a non-normal distribution, non-parametric statistics was applied. Quantitative variables were expressed with medians and Interquartile Range (IQR). In bivariate analysis, qualitative variables were compared using the χ^2^ test, and the quantitative ones with the Mann-Whitney U test. For significant variables, 95% CIs were established. For multivariate analysis, logistic and linear regression models were used. Variables finally included in the multivariate model were those showing statistical significance in the bivariate analysis or those with high clinical plausibility. The level of statistical significance was set to 0.05. Statistical analyses were performed using SPSS 25.0 (SPSS Inc., Armonk, NY, USA).

## Results

**Fig. 1:**
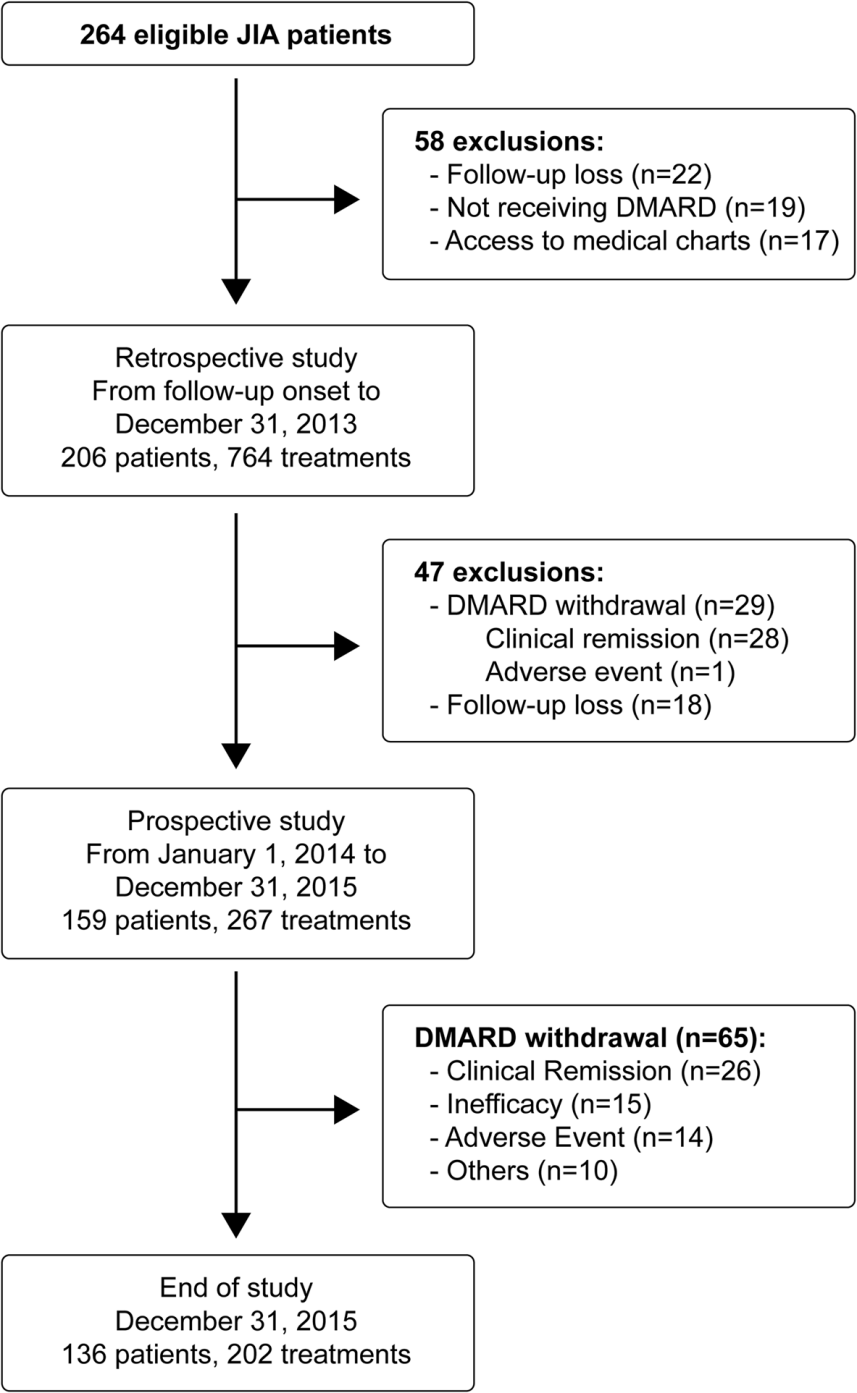
Cohort of JIA patients: retrospective and prospective studies. From follow-up onset to December 31, 2015

A total of 264 patients meeting the ILAR diagnostic criteria for JIA were included, of which 254 had received DMARD (synthetic or biologic). 206 patients were included in the retrospective study, which received 764 DMARD treatments. After 47 subsequent exclusions, 159 patients were included in the prospective study, which received 267 DMARD. At the end of follow-up, 136 patients remained on treatment, with 202 DMARD (Fig. 1).

The cohort was 71% female and 93% Caucasian, 52% had an oligoarticular JIA, 79% of which were persistent. Patients included had a median age at disease onset of 3 years [Inter quartile range (IQR) 4 years], at diagnosis of 4 years (IQR 5 years) and median disease duration at first DMARD onset of 4 years (IQR 8 years). Antinuclear antibodies (ANA) were detected in 68% of patients, and HLA B27 in 15% [67% of the enthesitis-related arthritis (ERA)]. Uveitis was present in 21% of patients (*n* = 43). Non-uveitis comorbidities were registered in 20% of patients (*n* = 41). Of these, 32% were other autoimmune diseases (*n* = 13), 32% genetic mutations or syndromes (n = 13), 10% chronic infections (n = 4), 10% congenital malformations (n = 4), and 16% miscellanea (*n* = 7). Thirty-two patients associated non-uveitis comorbidities with clinical significance (78%), being mild in 38% of the cases (*n* = 12), moderate in 34% (*n* = 11) and severe in 28% (*n* = 9).

Patients who achieved CR at least once throughout study follow-up (Table 1) were predominantly Caucasian (94%), had a persistent oligoarticular JIA (84%), did not associate non-uveitis comorbidities (87%), had not presented aprevious DMARD adverse event (75%), and had not required biologic DMARD (58%) (*p* < 0.05). When adjusting the analysis according to the JIA category, these results were confirmed for oligoarticular JIA. ERA patients achieved CR more frequently in the absence of non-uveitis comorbidities (88%, *n* = 15) (*p* = 0.035). The significant variables related to ever achieving CR obtained in the multivariate analysis were the absence of both non-uveitis comorbidities β 3.12 (CI 95% 1.5–6.51) (*p* = 0.002) and DMARD adverse event β 2.68 (CI 95% 1.42–5.07) (*p* = 0.002).

**Table 1:** Baseline characteristics of JIA patients according to ever achieving clinical remission of the disease

	Active Disease (***n*** = 62)	Clinical Remission (***n*** = 144)	TOTAL (***n*** = 206)	***p***
Female sex, n (%)	44 (71)	103 (72)	147 (71)	0.531
Caucasian ethnicity^a^, n (%)	55 (89)	136 (94)	191 (93)	0.016
JIA subtype n (%)				
Oligoarticular	24 (39)	83 (58)	107 (52)	0.285
Persistent^a^	14 (58)	70 (84)	84 (41)	0.016
Extended	10 (42)	13 (16)	23 (12)	
Poliarticular RF -	9 (15)	11 (8)	20 (10)	
Poliarticular RF +	2 (3)	5 (4)	7 (3)	
Psoriatic	6 (10)	11 (8)	17 (8)	
Systemic	4 (7)	13 (9)	17 (8)	
Enthesitis-related arthritis	13 (21)	17 (12)	30 (15)	
Undifferentiated	3 (5)	5 (4)	8 (4)	
No uveitis, n (%)	47 (76)	116 (81)	163 (79)	0.277
No comorbidities^a^, n (%)	40 (65)	125 (87)	165 (80)	0.001
Positive ANA, n (%)	36 (58)	103 (72)	139 (68)	0.067
Positive RF, n (%)	3 (5)	6 (4)	9 (4)	0.536
HLA B27 presence, n (%)	13 (21)	18 (13)	31 (15)	0.073
No AE with DMARD^a^, n (%)	30 (48)	108 (75)	138 (67)	0.001
No biologic DMARD^a^, n (%)	22 (36)	84 (58)	106 (52)	0.002

*n* Number, *JIA* Juvenile idiopathic arthritis, *RF-* Negative rheumatoid factor, *RF+* Positive rheumatoid factor, *ANA* Antinuclear antibodies, *HLA B27* Human leukocyte antigen, *AE* Adverse event, *DMARD* Disease modifying antirheumatic drug

^a^*p* < 0,05 Statistically significant differences using the χ^2^

### Retrospective study

Throughout the retrospective study, with a median follow-up period of 7 years (IQR 9 years), 764 treatments with DMARD were administered, 75% with sDMARD (*n* = 570) and 25% with bDMARD (*n* = 291).

Methotrexate was the sDMARD most frequently dispensed overall (*n* = 283), as the first (*n* = 169) and second (*n* = 57) sDMARD, and alone (*n* = 157). It was more commonly supplied to patients with oligoarticular subtype (*n* = 157), ANA positivity (*n* = 197) and uveitis (*n* = 63). Moreover, CR was the most frequent reason for its withdrawal (*n* = 82). Other sDMARD administered were Leflunomide (22%, *n* = 125), Tacrolimus (7%, *n* = 41), Cyclosporine A (7%, *n* = 39), Sulfasalazine (5%, *n* = 31), Azathioprine (4%, *n* = 24), Mycophenolate mofetil (3%, *n* = 15) and Hydroxychloroquine (2%, *n* = 12).

Etanercept was the most dispensed bDMARD overall (50%, *n* = 96), as first course (68%, *n* = 65), and alone (43%, *n* = 42). Other dispensed bDMARD were Adalimumab (42%, *n* = 55) Infliximab (10%, *n* = 20), Anakinra (5%, *n* = 9), Tocilizumab (*n* = 9) and others (*n* = 5).

Throughout the retrospective study, 64% of patients achieved CR at least once (131 of 206). The median number of times that patients achieved CR was 1 (range 1 to 4). A total of 199 treatments were administered amid CR status of the disease (199 of 764). When performing the analysis according to treatment group (Table 2), CR was more frequently observed in patients treated with sDMARD alone (53%). In this group, CR was associated with oligoarticular persistent subtype and Methotrexate treatment (*p* < 0.05). The absence of HLA B27 and DMARD toxicity were significantly related to higher rates of CR within all treatment groups (*p* < 0.05).

**Table 2:** Clinical remission in JIA patients treated with DMARD according to treatment group. Retrospective study results

	SDMARD alone ***n*** = 106 (53%)	SDMARD combined ***n*** = 37 (19%)	SDMARD BDMARD combined ***n*** = 48 (24%)	BDMARD alone ***n*** = 8 (4%)	Clinical remission ***n*** = 199	***p***
JIA ILAR category ^a^ n (%)						
Oligoarticular	66 (62)	18 (49)	25 (52)	3 (1)	112 (56)	0.003
Persistent	61 (92)	13 (72)	17 (68)	1 (33)	92 (82)	
HLA B27 absence ^a^ n (%)	89 (84)	33 (89)	25 (52)	6 (75)	153 (85)	0,023
Synthetic DMARD ^a^ n (%)						
Metotrexate	78 (74)	18 (49)	15 (31)	0	108 (54)	< 0.001
No AE with DMARD ^a^ n (%)	81 (76)	24 (65)	33 (69)	1 (13)	139 (70)	0.005
**Dose tapering*n*** **= 132**						
Dose tapering ^a^ n (%)	80 (75)	18 (49)	29 (60)	5 (62)	132 (66)	0,018
Tapering method n (%)						0,071
Interval increase	35 (44)	8 (44)	12 (41)	5 (100)	60 (45)	
Dose decrease	30 (37)	7 (39)	15 (31)	0	46 (35)	
Both	15 (19)	3 (17)	1 (3)	0	19 (14)	
Tapering duration, months, median (IQR)	15 (20)	14 (27)	21 (25)	40 (30)	16 (28)	0,190
Relapse amid tapering, n (%)	10 (13)	5 (28)	4 (14)	0	19 (14)	0,330
Withdrawal after tapering ^a^, n (%)	69 (86)	16 (89)	10 (34)	1 (13)	95 (72)	< 0,001
**Withdrawal due to CR*n*** **= 124**						
Withdrawal due to CR	71 (67)	29 (78)	21 (44)	3 (38)	124 (62)	0,728
Treatment duration to withdrawal due to RC ^a^, months, median (IQR)	31 (26)	42 (43)	34 (54)	68 (71)	31 (30)	0,037
Relapse after withdrawal n (%)	50 (70)	16 (55)	11 (52)	2 (67)	79 (64)	0,783
Time to relapse, months, median (IQR)	13 (25)	42 (41)	10 (18)	14	14 (32)	0,122

*SDMARD* Synthetic disease modifying drug, *BDMARD* Biologic disease modifying drug, *JIA* Juvenile idiopathic arthritis, *n* Number, *HLA B27* Human leukocyte antigen, *AE* Adverse event, *IQR* Interquartile range, *CR* Clinical remission

^a^*p* < 0,05 Statistically significant differences using the χ^2^ or Mann Whitney U tests

DMARD dose tapering was performed in 66% of administered treatments. It was more commonly observed in patients treated with sDMARD alone (75%) and combined with bDMARD (60%) groups (*p* = 0.018). No differences were observed regarding tapering method, order (sDMARD or bDMARD first), or duration [median 16 months, (IQR 28)]. Relapse amid dose tapering was scarce in all treatment groups (14%) (*p* > 0.05). DMARD withdrawal after dose tapering was significantly higher in patients receiving sDMARD exclusively, alone or combined (86 and 89%, respectively). No significant association was observed between CR regarding treatment group and disease duration at DMARD onset, the DMARD dispensed, or the order in which it was administered.

A total of 124 DMARD treatments were withdrawn due to CR of the disease (62%). Median treatment duration up to withdrawal was significantly shorter in patients receiving sDMARD alone (31 months, IQR 26 months). Moreover, of these, 64% relapsed after a median time of 14 months (IQR 32 months). Significantly lower relapse rates were observed in patients with persistent oligoarticular JIA (93%, *n* = 38) (*p* = 0.048) and in those in which DMARD dose was tapered before withdrawal (77%, *n* = 50) (*p* = 0.001). When performing the analysis according to JIA ILAR category, these results were confirmed for oligoarticular JIA.

Further analysis, adjusting by ILAR category was performed. Significant variables related to clinical remission in patients with oligoarticular forms of JIA are depicted in Table 3. CR predominated in patients receiving sDMARD alone (53%). Within this group, CR was more commonly observed in patients with persistent forms (92%), without associated comorbidities (88%), treated with MTX (80%) as first DMARD (67%), and when dose was tapered (77%) (*p* < 0.05).

**Table 3:** Clinical remission according to treatment group in patients with oligoarticular JIA

	SDMARD alone ***n*** = 66	SDMARD combined ***n*** = 18	SDMARD BDMARD combined ***n*** = 25	BDMARD alone ***n*** = 3	Clinical remission ***n*** = 112	***p***
Persistent	61 (92)	13 (72)	17 (68)	1 (33)	92 (46)	0.003
Comorbidities ^b^ absence, n (%)	58 (88)	15 (83)	23 (92)	1 (33)	97 (87)	0.042
Synthetic DMARD ^a^, n (%)						
Metotrexate	53 (80)	9 (50)	8 (50)	0	70 (35)	0.025
First DMARD course ^c^, n (%)	44 (67)	6 (28)	11 (44)	0	60 (54)	0.014
DMARD dose tapering ^a^, n (%)	51 (77)	8 (44)	15 (60)	3 (100)	77 (69)	0.024

*SDMARD* Synthetic disease modifying drug, *BDMARD* Biologic disease modifying drug, *JIA* Juvenile idiopathic arthritis, *n* Number;

^a^*p* < 0,05 Statistically significant differences using the χ^2^ test

^b^Comorbidities other than uveitis and not related to drug toxicity

^c^First DMARD course = DMARD dispensed in the first place, chronologically

Multivariate analysis demonstrated that variables significantly related to CR according to treatment group were persistent oligoarticular subtype (*p* = 0.001) and DMARD dose tapering (*p* = 0.002). These results were confirmed in patients with oligoarticular JIA when adjusting by ILAR category (*p* < 0.001).

At the end of the retrospective study, 18% of patients were in CR off treatment (*n* = 37). Of these, 28 were excluded due to maintaining remission and not requiring DMARD during the prospective study. A total of 236 DMARD were yet being administered, 60 of which were dispensed to patients in CR status of the disease. DMARD treatment continuation predominated in patients in CR receiving bDMARD, alone (63%) and combined with sDMARD (48%) (*p* = 0.001).

### Prospective study

Clinical remission was achieved by 17% of patients throughout the two-year follow-up prospective study (27 out of 159 patients). A total of 57 DMARD treatments were administered to patients in CR status of the disease (57 out of 267).

Patients demographic, clinical and treatment variables significantly related to CR, according to treatment group, are summarized in Table 4. Clinical Remission predominated in female patients in all treatment groups, except in the sDMARD and bDMARD combined group (*p* = 0.037). Persistent oligoarticular forms, ANA positivity, HLA B27 and uveitis absence were related to CR, particularly in patients treated with sDMARD alone (*p* < 0.05). When adjusting by JIA category, ANA association with CR was confirmed for oligoarticular and psoriatic subtypes and HLA B27 for oligoarticular and ERA subtypes in those patients treated with sDMARD alone (*p* < 0.05). Two variables were significantly related to CR according to treatment group in multivariable model: ANA (*p* = 0.034) and HLA B27 (*p* = 0.033). ANA negativity demonstrated a negative association with CR in patients treated with sDMARD alone β − 1.59 (CI 95% 0.43–0.955) (*p* = 0.044).

**Table 4:** Clinical remission in JIA patients treated with DMARD according to treatment group. Prospective study results

	SDMARD alone ***n*** = 33 (43%)	SDMARD combined ***n*** = 6 (8%)	SDMARD BDMARD combined ***n*** = 23 (30%)	BDMARD alone ***n*** = 14 (18%)	Clinical remission ***n*** = 76	***p***
Female sex ^a^, n (%)	24 (73)	4 (67)	8 (35)	9 (64)	45 (59)	0.037
Persistent oligoarticular ^a^, n (%)	19 (91)	6 (100)	4 (57)	2 (50)	31 (82)	0.048
ANA positivity ^a^, n (%)	26 (79)	6 (100)	12 (52)	6 (43)	50 (66)	0.013
HLA B27 absence ^a^, n (%)	31 (94)	0	12 (52)	8 (57)	57 (75)	0.005
Uveitis absence ^a^, n (%)	27 (82)	2 (33)	21 (91)	13 (93)	63 (83)	0.006
Biologic DMARD ^a^, n (%)						
Etanercept	0	0	5 (36)	10 (71)	15 (54)	0.040
DMARD dose tapering ^a^, n (%)	29 (88)	3 (50)	14 (61)	11 (79)	57 (75)	0.050

*SDMARD* Synthetic disease modifying drug, *BDMARD* Biologic disease modifying drug, *JIA* Juvenile idiopathic arthritis, *n* Number; *ANA* Antinuclear antibodies, *HLA B27* Human leukocyte antigen, *AE* Adverse event; *IQR* Interquartile range, *CR* Clinical remission

^a^*p* < 0,05 Statistically significant differences using the χ^2^ or Mann Whitney U tests

DMARD dose was tapered in 74% of treatments, with median of two reductions per treatment (range 1 to 5). Dose tapering predominated in patients treated with synthetic or biologic DMARD administered alone (88 and 79%, respectively) (*p* = 0.050). No differences in method, order, or duration (median 24 months, IQR 29) were observed among treatment groups. Relapse throughout dose tapering was scarce (11%) and 39% of DMARD treatments were subsequently withdrawn, showing no significant differences regarding treatment (*p* > 0.05). Out of 267 treatments administered, 34% were withdrawn due to CR of the disease (*n* = 26) with median treatment duration to withdrawal of 12 months (IQR 11) and 12% of relapse (*n* = 3). Median time to relapse was 10 months, comparable in all treatment groups (*p* > 0.05).

To conclude, when the follow-up was completed, 70% of the patients in the cohort had attained CR at least once (144 out of 206), and 29% were in CR off all medication (59 out of 206). Of these, 69% achieved CR off medication throughout the retrospective study and maintained it along the 24-month prospective study (*n* = 41). Clinical remission off medication (*n* = 59), regarding JIA subtype, was attained by 30 oligoarticular, 11 ERA, 10 systemic, 4 poliarticular RF negative, 3 undifferentiated and 1 poliarticular RF positive patients.

## Discussion

Clinical remission of the disease, based on Wallace criteria, was achieved at least once by 70% of the JIA patients included in the cohort along a median of 9-year follow-up, and was the main reason of DMARD withdrawal in both studies.

Greater CR rates were observed along retrospective study, which might be explained by the longer follow-up duration. In accordance with this observation, two studies have hypothesized, based on study results observed throughout last decade, that CR on medication increases with longer disease duration [9, 17].

In our study, patients were predominantly Caucasian females, with early JIA onset (< 5 years), oligoarticular persistent JIA, ANA positivity and early age at DMARD onset (< 6 years). MTX was the most frequently administered sDMARD and the most commonly administered alone.

Ever attaining CR was related to persistent oligoarticular subtype, absence of coexisting comorbidities (other than uveitis) and not having undergone an adverse event with DMARD therapy. These results were confirmed for oligoarticular JIA patients, among which CR predominated in those treated with Methotrexate alone and as first DMARD. Patients with oligoarticular JIA, particularly those with persistent subtype, have achieved the highest CR rates on and off medication in various studies, being treated in most cases with MTX as DMARD of choice [17, 25–27].

On the other hand, coexisting comorbidities in JIA patients, including other autoimmune diseases, genetic syndromes, or chronic infections, among others, could lead to both: a higher occurrence of DMARD toxicity and inefficacy. Raab et al. studied associated comorbidities in young adult JIA patients of the JuMBO register and concluded that comorbidities have a significant impact on disease activity and global health status [28]. Furthermore, Simon et al. have recently put in evidence that patients with JIA are more likely to have concurrent autoimmune diseases, and this association might have an important role in JIA treatment decisions and outcomes [29].

Lastly, the negative influence of DMARD toxicity in ever achieving CR in JIA patients could be explained in those having a more aggressive course of the disease. In these, higher DMARD doses and therapeutic targets are combined, leading to a greater likelihood of DMARD withdrawal due to drug toxicity and subsequent poorer control of disease activity.

According to therapeutic approach, CR was observed in patients treated with synthetic DMARD alone more than half of the times, being Methotrexate the most administered sDMARD and being CR the predominant reason of its withdrawal. These findings support that Methotrexate is still the DMARD of choice in most JIA categories, even in the biologic era [10, 30, 31]. Within this group CR was associated with female sex, persistent oligoarticular subtype, absence of comorbidities (other than uveitis) and Methotrexate treatment as first DMARD course. ANA positivity, the absence of HLA B27, uveitis and DMARD toxicity, and having performed DMARD dose tapering were related to higher CR rates within all treatment groups, but more significantly to patients treated with sDMARD alone. Furthermore, ANA association with CR was confirmed for oligoarticular and psoriatic subtypes and HLA B27 for oligoarticular and ERA subtypes in those patients treated with sDMARD alone. However, the results must be considered cautiously, since the number of patients in some categories was scarce.

The role of ANA positivity in the likelihood of achieving and sustaining CR, and therefore, in short and long-term outcomes, has not yet been clarified. Some studies suggest that ANA negativity would lead to a poorer response to MTX throughout the first 6 months of the disease, a higher active joint count along the first 6 to 24 months, regardless on active joint count, and to a persistent active disease through the first 3 years from JIA onset [25, 32–34]. The possible association between ANA positivity and a better MTX response would explain that oligoarticular patients included in the study, and not having coexistent comorbidities (uveitis or others), would have solely required treatment with Methotrexate alone to attain CR. These results would support what has been recently suggested in two studies which showed that JIA patients with early onset, ANA positivity and lower active joint count, constitute a homogeneous JIA subtype with less aggressive course of the disease, higher likelihood of achieving CR and subsequently better outcome [35, 36]. Nevertheless, our results contravene the results obtained in two other studies which suggest that ANA status does not alter remission rates nor JIA outcome [37, 38].

Regarding HLA B27, several studies have put in evidence that HLA B27 presence in JIA patients leads to a higher risk of bone erosions and radiologic structural damage appearance [39, 40], a more persistent and extended course of the disease [41, 42], and a lower likelihood of achieving clinical remission [12, 25].

Dose tapering was performed in two thirds of patients in CR on medication. Relapse throughout dose tapering was scarce in both studies. Two thirds of DMARD treatments administered along CR of the disease were withdrawn throughout our retrospective study, with a subsequent relapse observed in two thirds of the cases, after a median time to relapse of about 12 months with comparable results in all treatment groups. Significant lower rates of relapse after withdrawal were observed in patients with persistent oligoarticular JIA, treated with sDMARD alone (mostly Methotrexate) in which the drug dose was previously tapered. Nonetheless, no differences in method, order (sDMARD or bDMARD first) or duration (median longer than 12 months in both studies) of dose tapering were observed within treatment groups in both studies, and neither was associated with CR of the disease nor relapse after withdrawal. Regarding patients in CR on medication, our therapeutic strategy is comparable to the one performed by most rheumatologists participating in two survey studies, who would wait at least 12 months while in CR on medication to start considering DMARD tapering or withdrawal [14, 15]. Furthermore, given that CR was more frequently achieved in patients treated with MTX alone in our study, these results would support that maintaining MTX at least for 12 months after achieving CR before withdrawal is associated with a higher likelihood of maintaining remission off treatment, as suggested by Klotsche*et al* [43]. One of the limitations of our study is that the exact date of clinical remission start was unavailable in most patients, and therefore, total duration of CR on treatment could not be measured. Duration of CR on medication remains currently of significant interest given that its association with relapse after treatment discontinuation has shown conflicting results [13].

When the study finalised after a median follow-up time of 9 years, more than two thirds had attained CR at least once, and nearly one third of patients were in clinical remission off all medication, which is slightly lower than the 42% observed in Nordalet al. study [44]. Considering that in our study persistent oligoarticular JIA is the most prevalent JIA subtype, we might have underestimated CR rates through follow-up losses, given that patients lost to follow up would more likely be in remission than those who maintain it.

On the other hand, two thirds were still on DMARD therapy. It should be noted that in those patients treated with sDMARD and bDMARD combined, tapering duration and number of times the dose was tapered were greater, whereas subsequent withdrawal and following flares were less frequently observed in the prospective study. This fact would support the recently growing belief that in some patients, especially those with persistent and extended poliarticular RF positive, systemic or ERA JIA, the disease behaves more aggressively, needing more specific therapeutic targets and a sustained treatment over time, even when CR is achieved. In those scenarios, maintaining a minimum and progressively tapered dose for at least 12 months (before even considering withdrawal) would appear to be a good therapeutic approach to minimize subsequent relapses.

The main limitation of our study is its observational mono-centric and non-controlled design. This fact might have led to selection bias, having a lower number of patients included in a predominantly oligoarticular JIA cohort. Consequently, the depicted results should be applied cautiously to other JIA categories. That being said, the coincidence of the results obtained in both retrospective and prospective studies grants coherence to the conclusions provided. Lastly, this study fails to describe total duration of CR both on and off medication as the exact date of CR onset was unavailable in most patients. Clinical remission’s survival in JIA patients remains an issue of great concern given the complexity of establishing and defining when CR itself begins.

## Conclusions

More than two thirds of JIA patients attained CR along the 9 years of follow-up, and nearly one third achieved CR off medication. Females with early JIA onset, lower active joint count and ANA positivity were the ones achieving and sustaining remission more frequently, especially when receiving synthetic DMARD alone and in the absence of HLA B27, comorbidities or previous DMARD toxicity. In patients who already have attained remission, a progressive DMARD dose tapering for at least 1 year before considering withdrawal would minimize the probability of subsequent relapse, particularly in those who required a biologic DMARD to achieve a quiescent state of the disease.

## Data Availability

The datasets used and/or analysed during the current study are available from the corresponding author on reasonable request.

## Abbreviations

JIA: Juvenile Idiopathic Arthritis
CR: Clinical Remission
DMARD: Disease Modifying Antirheumatic Drugs
ANA: Antinuclear Antibodies
RF: Rheumatoid Factor
HLA: Human Leukocyte Antigen
ERA: Enthesitis Related Arthritis
IQR: Interquartile Range
MTX: Methotrexate
